# Strengthening maternal healthcare in Ghana: utilizing the community-based health planning and services model as a vehicle

**DOI:** 10.3389/fgwh.2025.1590452

**Published:** 2025-09-12

**Authors:** Linus Baatiema

**Affiliations:** 1 Ghana Health Service, Upper West Regional Health Directorate, Wa, Ghana; 2 Centre for Migration, Security and International Relations, Faculty of Public Policy and Governance, Simon Diedong Dombo University of Business and Integrated Development Studies, Wa, Ghana; 3 Department of Public Health, L&E Research Consult Ltd., Wa, Ghana

**Keywords:** CHPS, maternal healthcare, primary healthcare, community involvement, community health officers, community health management committees and community health volunteers (CHVs)

## Abstract

**Introduction/aim:**

This review explores the Community-Based Health Planning and Services (CHPS) model and its impact on maternal healthcare delivery in Ghana's primary healthcare system. It highlights both the achievements and challenges of the initiative, focusing on community involvement, trained human resources, and effective referral linkages.

**Aim:**

To critically examine how CHPS model contributes to strengthening maternal healthcare delivery in Ghana, by assessing its successes, challenges, and potential for promoting equitable and sustainable health outcome.

**Methods:**

The study systematically reviewed literature from databases including PubMed, Google Scholar, and JSTOR, using keywords related to CHPS, maternal and child healthcare, reproductive health, and community health initiatives. Peer-reviewed articles, reports, and grey literature published within the past 10 years were prioritized, with additional insights drawn from references in the selected studies. The thematic areas were organized into maternal and child health services, reproductive health challenges, community health programs, and CHPS's role in addressing healthcare inequalities.

**Results:**

Findings reveal that, CHPS as a strategy has significant potential to improve maternal health outcomes, reduce mortality rates, and ensure equitable access to care for women in rural and underserved areas. Key challenges such as inadequate funding, staff shortages, political interference, and cultural barriers persist, limiting the model's overall impact.

**Conclusion:**

The study provides policy recommendations to enhance the effectiveness of the CHPS model and calls for a collective effort from health sector stakeholders to build a sustainable maternal healthcare system in Ghana.

## Introduction

Maternal health is one of the most important aspects of public health as it seeks to enhance the status of mothers and newborns. Despite the great improvement in the past decades, maternal morbidity is still a one of the top global public health concern, particularly in low and middle-income countries (1). Statistics global also pointout that, 287,000 maternal deaths occur each year, with Sub-Saharan Africa accounting for 70% of deaths (1). Many of these deaths are avoidable and are concentrated in the poorer regions of the world as timely access to high quality maternity care is unavailable (2–4). Maternal health is a fundamental component of the Primary Health Care (PHC) system, as women require medical care during pregnancy, childbirth, and the postnatal period (5–7).

The PHC system is important for the appropriate distribution of key maternal services, such as antenatal care, attendance during childbirth, and postnatal care. Nonetheless, especially in low-resource contexts, the PHC system has more work than proportionate resources, and is thus poorly equipped with the infrastructure, human resources, and medical supplies to assist pregnant women, more so in the rural areas (8, 9).

Maternal health policies have also been enacted and strengthened at the primary level of health systems in countries. The World Health Organization and other countries have developed initiatives such as the Global Strategy for Women, Children and Adolescents' Health (2016–2030) or SDGs, particularly target 3.1 which intends to have less than 70 maternal deaths for every 100,000 live births by the year 2030 (10). Such policies, particularly in low-income countries, have also promoted the organization of maternal health services in the context of primary health care with an emphasis on mobilizing communities, task-shifting and positioning health workers in rural areas (11). This way, the healthcare system will become less centralized so that maternal healthcare service provision is enhanced, leading to better health results for mothers and their infants, including reductions in maternal mortality.

In Ghana, the Community-based Health Planning and Services (CHPS) initiative represents a key policy framework aimed at strengthening maternal healthcare at the primary level (12, 13). CHPS was introduced to address inequities in healthcare delivery, particularly in rural and underserved areas, by providing decentralized healthcare through community health compounds staffed by Community Health Officers (CHOs). These officers offer essential maternal and child health services, including antenatal care, skilled deliveries, and postnatal care (12, 14, 15). CHPS compounds have significantly increased access to maternal health services, particularly in remote regions where women previously faced significant barriers to care.

The essence of this study is to explore how leveraging CHPS can further strengthen maternal healthcare at the primary healthcare level. While the CHPS initiative has had a positive impact, challenges such as insufficient staffing, inadequate medical supplies, and sociocultural barriers continue to hinder its full potential. This study aims to analyze the role of CHPS in maternal healthcare delivery, identify gaps in service provision, and propose recommendations for enhancing the initiative's effectiveness in reducing maternal mortality and improving maternal health outcomes in resource-limited settings.

## Literature search strategy and sources

The literature search procedure was conducted systematically across multiple databases, including PubMed, Google Scholar, and JSTOR. Various keywords related to CHPS and maternal and child healthcare, reproductive health, and community health initiatives were used to find peer-reviewed articles, reports, and grey literature in the databases. The search was limited to articles published within the past 10 years to incorporate the most recent findings and data. Besides, relevant studies cited in the selected articles were reviewed and added. However, it did not limit the review since it included other diverse sources with supporting evidence, making the literature review more balanced and comprehensive in informing the study's eye and approach. The thematic areas in the literature were organized into the main sections, for example, maternal and child health services, reproductive health challenges, community health programs, and the role of the CHPS in addressing inequalities in health care.

### Overview of the CHPS model in Ghana

In Ghana, the CHPS initiative seeks to revolutionize the healthcare delivery in resource limited areas. It was introduced sometime in the 1990's and the CHPS initiative being a decentralized healthcare delivery design advocates for community participation and uses basic health workers at the grass roots to render basic health care (16). The basic aim of implementing CHPS is to increase access to PHC, especially maternal and child health care in those regions which are constrained by geographical and resource limitations to accessing conventional health care services.

### Policy genesis and evolution

The CHPS model has its roots in a trial carried out by the Navrongo Health Research Centre in the Upper East Region of Ghana, which aimed to investigate low-cost and sustainable means of providing primary healthcare in rural areas (14). This has been referred to as the Navrongo Experiment which proved that health outcomes, especially those concerning mother and child health could be improved if health workers were trained and sent to those specific communities. This success experienced with Navrongo model necessitated that the CHPS model to be adopted across the country and that's why the Ministry of Health and Ghana health service (GHS) in 1999 accepted it as national policy (14, 17).

Consequently, CHPS was included as part of national health sector policies, the National Health Policy and the Ghana Poverty Reduction Strategy, as an approach to enhancing health indices in the rural population (17, 18). The framework delivers optimally where there is community participation, where the community is involved in the planning, implementation and monitoring of health services. One of the crucial aspects of the CHPS model is the deployment of Community Health Officers (CHOs), who are trained community-based health workers. These officers work in community health compounds (CHCs) and are given the responsibility of delivering primary health care services, including usage of maternal and child health, family planning, immunization and control of diseases.

To address inequities in healthcare access, particularly in rural regions, CHPS was designed to decentralize healthcare services by establishing community health zones, staffed by trained Community Health Officers (CHOs) who reside within the communities they serve (14). CHPS compounds are vital for delivering maternal healthcare, including antenatal care (ANC), skilled deliveries, and postnatal care (PNC). These units bridge the gap between the formal health system and the community, ensuring healthcare reaches women who would otherwise face geographical or financial barriers to accessing care. By involving communities in health planning and service provision, CHPS enhances local ownership and accountability in healthcare delivery (19).

### Policy and pratice

At the national level, the Ministry of Health (MoH) and the Ghana Health Service (GHS) provide overarching policy and strategic direction for CHPS, ensuring alignment with national health priorities such as the Safe Motherhood Initiative and the Sustainable Development Goals (SDGs). This level focuses on mobilizing resources, developing policies, and coordinating donor activities to enhance maternal healthcare delivery across the country. At the regional level, health directorates translate these policies into actionable programs, overseeing the training and supervision of Community Health Officers (CHOs) and ensuring consistency in maternal healthcare services like antenatal care and safe deliveries. The district level, through District Health Management Teams (DHMTs), plays a critical role in resource mobilization and the direct implementation of CHPS activities, including managing health personnel and ensuring that maternal health programs are effectively executed (20). Lastly, at the sub-district level, healthcare teams focus on community engagement and service delivery, collaborating with local actors to tailor maternal healthcare services to the specific needs of the community. This level fosters trust between healthcare providers and communities while ensuring effective referral systems for complicated pregnancies, linking CHPS zones to higher-level healthcare facilities (14, 21, 22).

## Role of community health officers (CHOs) and community actors in strengthening maternal healthcare

### Role of CHOs

These Community Health Officers (CHOs) are a sine qua non of the Community-Based Health Planning and Services (CHPS) initiative, especially in the rural, hard to reach areas with prominent health care access problems. They are the first contact healthcare workers with the responsibility of providing complete maternal health care service which includes antenatal care, skilled delivery, postnatal care and family planning. In Ghana, pregnant women's antenatal care coverage significantly increased from 91% in 2006 to 96% in 2021 showing the importance of the services offered by CHOs (23). The early recognition of potential complications in pregnancy is among their many critical and indispensable roles. CHOs are orientated on assessing risks, and symptoms such as preeclampsia or obstructed labor, so that problematic pregnancies can be referred to higher levels of care. Ghana Health Service states that maternal mortality has improved partly because appropriate referrals are made by CHOs to other institutions; that of maternal mortality rate decreased from 319 deaths per 100,000 live births in 2015 to 308 per 100,000 live births in the year 2020 (1).

CHOs beyond providing clinical care also embark on health education of the communities on a regular basis. These campaigns address the common cultural and social factors that inhibit women from utilizing the maternal health care services. For example, it has been reported that in some areas, women are discouraged from going to the health facilities because they believe in traditional practices. Being in the community as one of them, CHOs, do away with such misconceptions and promote health seeking behavior. Studies have reported improved rates of skilled deliveries due to health education activities conducted by CHOs. This assertion is affirmed by the statistic of skill delivery in the 2022 Ghana Demographic and Health Survey (GDHS) data which posit that skilled birth attendance continued to improve, with the national average rising to approximately 79%. This indicates steady progress from the 74% reported in 2014. The improvement reflects Ghana's ongoing efforts to strengthen primary healthcare, particularly through the expansion of Community-based Health Planning and Services (CHPS) compounds and increased accessibility to maternal health services. These advancements have contributed to better maternal and neonatal health outcomes across the country.

### Role of community actors

Health outcomes at the community level, especially in relation to maternal health, are considerably improved with the involvement of community actors such as Community Health Management Committees (CHMCs), Traditional Birth Attendants (TBAs), and Community Health Volunteers (CHVs). CHMCs work hand-in-hand with Community Health Officers (CHOs) to ensure that health delivery services meet the demands of the people. They are active participants in shaping programs that affect maternal health, making sure that health needs in the community including maternal health are properly catered for. Community involvement has been evaluated and proven to enhance maternal health care services. Where maternal health committees and community health management committees are active, the uptake of maternal health programs is much higher as compared to areas with no active communities' involvement (24, 25).

In the past, maternal healthcare services in the rural established communities were solely provided by Traditional Birth Attendants (TBAs), who now work alongside Community Health Officers (CHOs). In the meantime, TBAs are referring pregnant women to CHPS facilities for skilled care, which also aids in the merging of the traditional and modern healthcare systems. This model has worked quite well in areas where there is a high cultural dependency on TBAs. For instance, in the Upper East Region, TBAs now assist in bringing women to a CHPS center, thus there was a 15% improvement in health facility delivery from the year 2017 to the year 2020 (26). Community Health Volunteers (CHVs) have also been instrumental in helping extend maternal health services. They engage in outreach and information sessions encouraging women to visit the health facilities for antenatal care and deliveries. The records from the Ghana Health Service suggest that CHV-managed outreach program increased antenatal clinic attendance by 12% in the designated rural CHPS areas (21). There are also maternal healthcare advocates among the community; these include religious leaders and traditional rulers.

## Contribution of CHPS to maternal healthcare

The Community-Based Health Planning and Services (CHPS) initiative represents a transformative model for delivering primary healthcare in Ghana, with a particular emphasis on maternal and child health. Since its inception in the late 1990s and formal national rollout in 2000, CHPS has sought to address geographical and socio-economic barriers to healthcare access, especially for rural populations where maternal health outcomes have historically lagged. CHPS compounds, staffed by trained Community Health Officers (CHOs), provide essential services such as antenatal care (ANC), skilled delivery, postnatal care, and family planning at the doorstep of communities. This decentralized model has strengthened early pregnancy identification, promoted facility-based deliveries, and fostered trust between health workers and communities.

**Figure 1:**
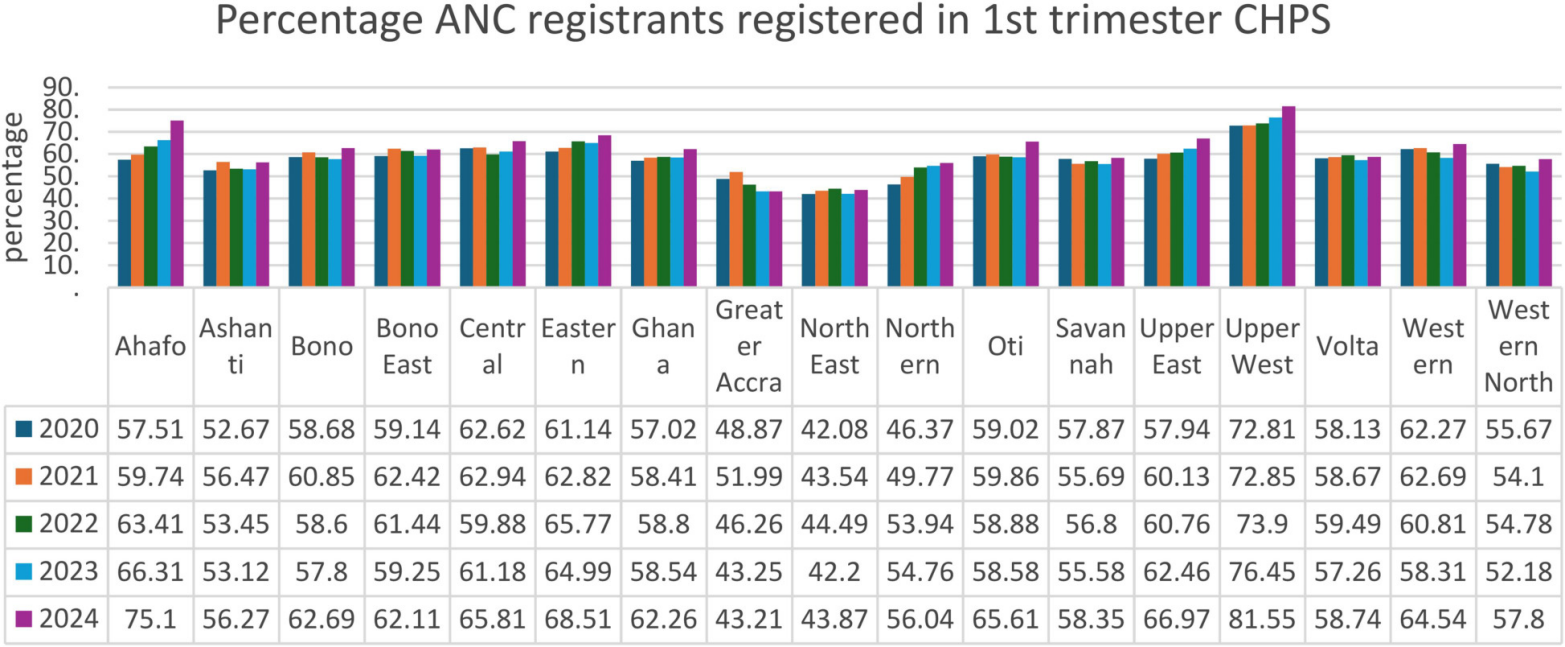
ANC registrants registered in 1st trimester CHPS.

**Figure 2:**
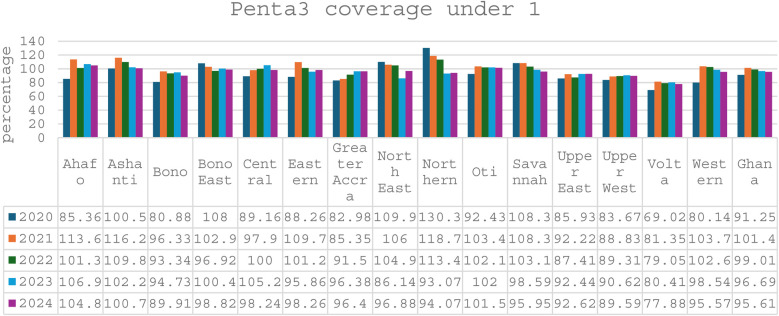
Penta3 coverage under 1.

Evidence shows that the CHPS initiative has made significant contributions to improving maternal healthcare indicators over time. Between 2020 and 2024, the proportion of pregnant women registering for ANC in the first trimester at the national level increased from 57.02% to 62.26%, a vital step for early detection and management of pregnancy-related complications (see Figure 1). CHPS-reliant regions such as the Upper West and Upper East recorded even higher improvements, with the Upper West rising from 72.81% to 81.55% and the Upper East from 57.94% to 66.97%, reflecting the impact of strengthened community-based service delivery systems (DHIMS 2, Ghana Health Service, 2024). These improvements reflect the proactive community engagement and household outreach strategies embedded within the CHPS model. In addition, CHPS compounds have played a pivotal role in improving immunization coverage for children, which indirectly benefits maternal health by encouraging regular healthcare interactions. National Penta 3 coverage, for example, improved from 91.25% in 2020 to 95.61% in 2024, (see Figure 2) highlighting the resilience and reach of CHPS services even during challenging periods such as the COVID-19 pandemic (DHIMS 2, Ghana Health Service, 2024).

**Figure 3:**
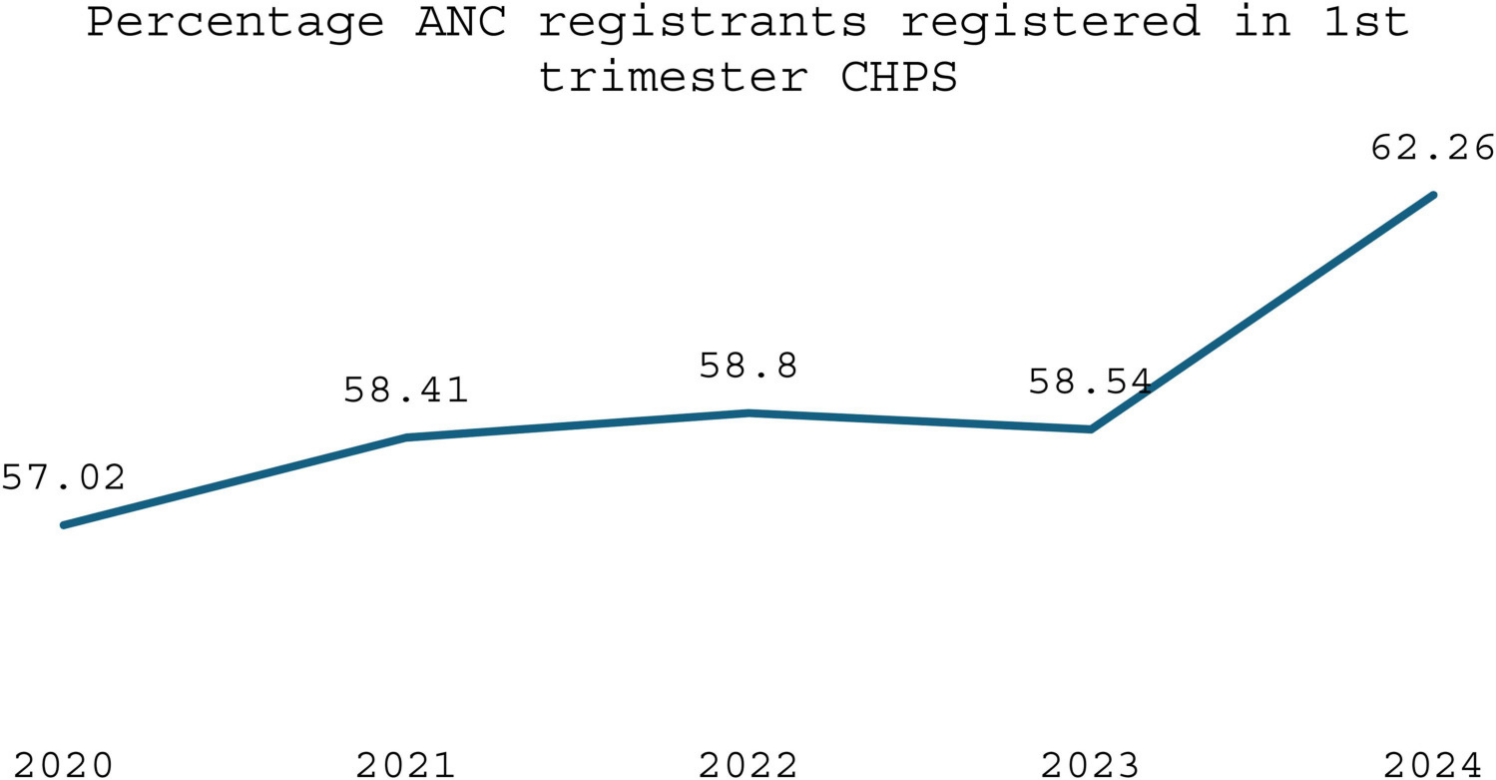
Trend of ANC registrants registered in 1st trimester CHPS.

**Figure 4:**
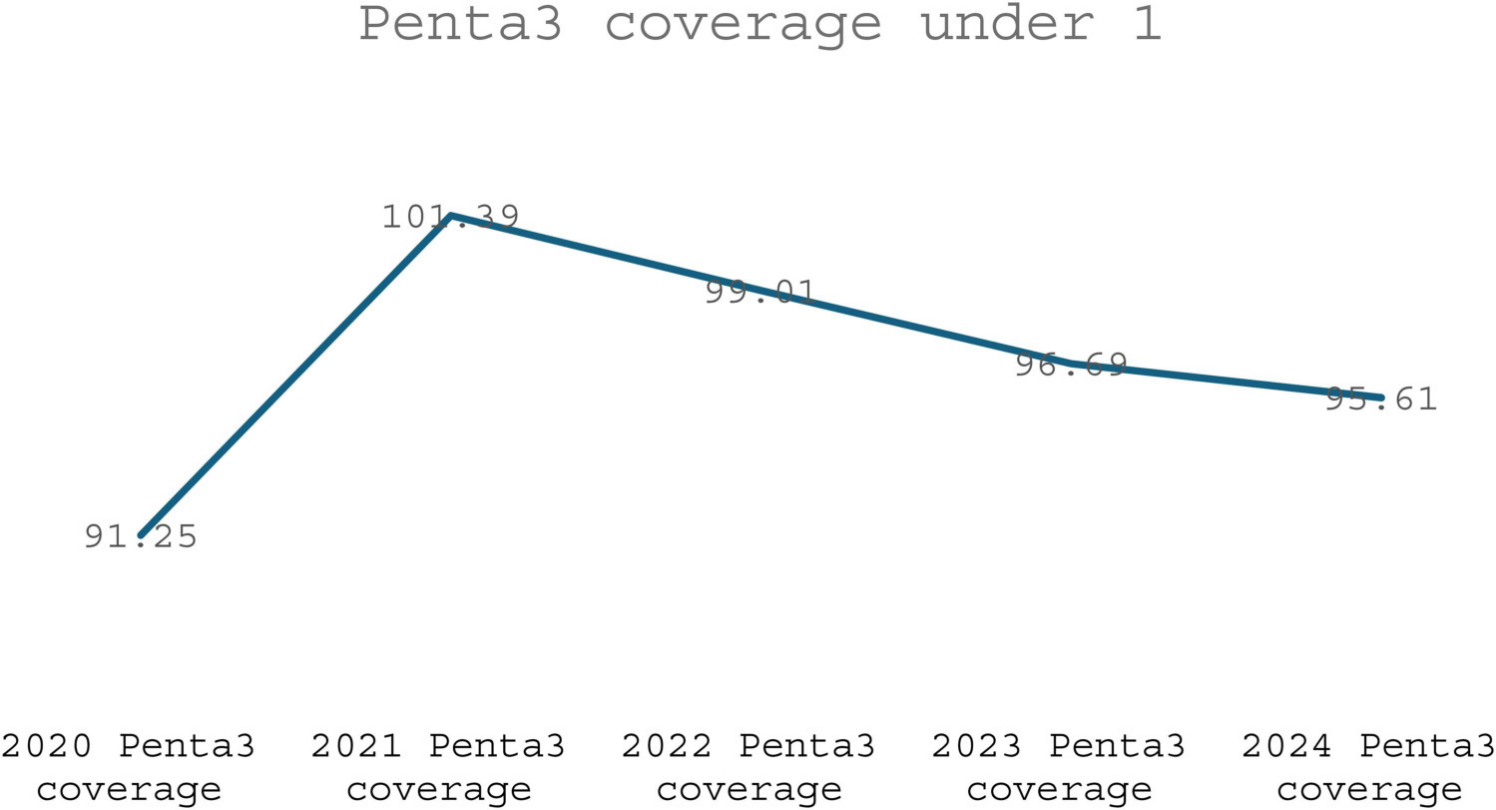
Trend of Penta3 coverage under 1.

Despite these achievements, CHPS faces ongoing challenges that limit its full potential. Nevertheless, the overall contribution of CHPS to maternal healthcare is undeniable, positioning it as a critical vehicle for achieving Ghana's maternal health targets under the Sustainable Development Goals (SDGs). Strengthening the CHPS system through targeted investments, policy support, and community empowerment remains essential to further advance maternal health outcomes, particularly for the most vulnerable populations (see Figures 1–4).

## Challenges and gaps in CHPS-led maternal healthcare

In spite of the laudable success brought about by the Community-Based Health Planning and Services (CHPS) model of maternal health care in Ghana, there are still several challenges and gaps that dampen its full implementation. Top on the list is lack of enough finances, which limits the provision of basic medical supplies, equipment and medical workers who are necessary for quality maternal care. Many of these CHPS compounds are not well equipped with the requisite facilities such as operational delivery suites, medical commodities motorbikes, or even emergency rescue services for referrals. According to (14) approximately 45% of the delivery kits and supplies in use in rural CHPS areas are below the recommended standards which has adversely compromised the maternal healthcare services offered in such areas.

Additionally, another major obstacle is the unavailability of personnel. A shortages of health workers, especially trained Community Health officers (CHOs) is particularly severe in rural regions, which are some of the the poorest counties to have limited access to healthcare. The situation is even more critical if the demand is for trained CHOs as they are the very ones responsible for offering maternal health services at CHPS. Some CHPS zones are very understaffed, owing to financial capping or restrictions on recruitment, and many healthcare workers avoid working in rural areas. Even (27) acknowledges that some of the zones assign one CHO multiple communities thereby stretching their services thin and making it impossible for them to attend to mothers continuously. This concern is aggravated by the fact that most CHOs who are available are often overworked, leading to extreme fatigue which hampers their efficiency and the quality of health care provided.

Furthermore, there are concerns regarding the member of staff turnover and the undue influence of politics in the posting of health workers. In most cases, the health care needs of a given area may not be the sole determinant of the health care personnel employment especially in the remote and developing areas. This leads to a variation in the number of health care workers available or sent to work in various regions where trained personnel are sent to urban or more developed regions, while the rural and less developed free CHPS zones remain with very few or no workers. Political influence in posting can work against the CHPS model because the health care workers are often not willing to accept the assignment in the rural CHPS compounds thus affecting the provision of maternal health services in these regions (28, 29).

In addition, a large number of sex health service providers post to the northern regions of Ghana is because a lot of the health workers are not willing to be posted to CHPS zones. The belief that infrastructural facilities, including basic social services are usually absent in these areas such as availability of electricity, running water, and decent housing prevents health personnel from effective delivery of services in such regions. This view adds to the already dire threats of healthcare staff retention, since healthcare workers are less likely to be posted in regions with urban centres as outreach regions, but rather human resources directed at urban health centres or better facilities. Most health professionals were Wastage of national resources in recruiting and training healthcare staff has been a disturbing realization as rural areas are often considered to be punishment zones, and this affects the recruitment and retention of personnel in CHPS zones (21).

The referral system in the CHPS model also faces significant challenges. CHOs are trained to identify high-risk pregnancies and refer them to higher-level health facilities, but the lack of reliable and affordable transport, coupled with poor road infrastructure in rural areas, makes timely referrals difficult. This gap in the referral system often results in delays in accessing specialized care, contributing to the high maternal mortality rates in some rural areas.

## Innovative approaches and best practices to mitigating maternal healthcare at the primary healthcare level

Creative approaches have been developed to overcome some of the challenges that CHPS faces in delivering maternal healthcare. For example, in certain areas of the country, mHealth technologies have been deployed to enhance the communication between the CHOs and health facilities, in turn facilitating improved monitoring of maternal health parameters. SMS-based reminders for ANC appointments, below, for example, has improved ANC attendance in some CHPS zones (12).

As the maternal health landscape evolves, the continue training and capasity building of CHOs becomes essential to ensure high-quality maternal healthcare. Maternal health outcomes depend greatly on the continuing education and skills enhancement of CHOs, midwives, and community workers. In several districts, funded maternal health services were sustained through the strategic alliance of CHPS units with Non-Governmental Organizations (30). These partnership, often supported by donor funding, have enabled CHPS zones to assess the resources needed to deliver more comprehensive care. Such collaboration hightlight the importance of external funding and cross-sectional partnership in ensuring the long-term success of maternal health programs at the community level.

Strengthening referral systems is crucial, requiring efficient pathways and collaboration with local transport providers to ensure timely transfers for high-risk pregnancies. Community engagement initiatives should be enhanced by involving local leaders, Community Health Management Committees (CHMCs), and Traditional Birth Attendants (TBAs) in educational campaigns that address cultural barriers. Establishing a robust monitoring and evaluation framework will facilitate ongoing assessment of maternal health outcomes, while advocacy for supportive policies and clear staffing guidelines will minimize political interference. Strengthening partnerships between CHOs and community actors, alongside leveraging technology such as mobile health applications, will improve service delivery and access to information, ultimately reducing maternal mortality and improving health outcomes for women in rural Ghana. According to (14), mHealth interventions play a paramount role in supporting pain management, relaxation strategies, facilitating decision-making, tracking maternal and foetal health, providing postpartum education and support, breastfeeding assistance, maternal well-being enhancement, infant health promotion, mental health support, and parental guidance.

The Community Scorecard (CSC) approach, also referred to as social accountability monitoring tool has become more innovative and contributed significantly towards accountability, transparency, and engagement of the community in maternal health services. The CSC allows for the inclusion of the community members, health workers, and other relevant stakeholders in the evaluation of healthcare services and thus seeks to fill the gap through a process of participatory problem solving. To illustrate, in the case of CHPS zones, the CSC can assess maternal health services and most members of the community can raise their issues concerning health service provision, resources, and local cultures. In addition, such a participatory feedback mechanism enhances the relationship of the healthcare providers and the community, which, in returns increase the trust and the maternal health care services provision. It also allows health-care administrators and policy makers to assess the delivery of services and design subsequent strategies using the information obtained from the CSC.

The Quality Care Model has managed to grow maternal health services at the base level of primary care, especially in the CHPS battle, by tackling issues of access, capacity, and community support. In doing so, the Model has helped to ensure that more women, especially those in the rural areas, are able to access much needed maternal health services, by addressing the issue of care access. The care of community population has also improved with increase in a number of community health officers (CHOs) who have been actively recruited and trained as human resources investments. In addition, continuous training and capacity-building of CHOs, midwives and other health workers have enabled them to manage pregnancy complications and provide antenatal and postnatal care as well as community education on maternal health. All in all, the model has been associated with improved vertical health care services in primary care, driving improvements in maternal health and mortality indicators in the community.

To enhance CHPS-led maternal healthcare in Ghana, policymakers should allocate increased financial resources specifically for CHPS programs, prioritizing regions with high maternal mortality rates. Targeted recruitment and retention strategies for Community Health Officers (CHOs) should be developed to encourage placements in rural areas, supported by financial incentives and continuous training opportunities.

## Limitations and future directions

The review offers a comprehensive exploration of the CHPS model and its impact on maternal healthcare in Ghana. Its strengths lie in the thorough analysis of the model's historical evolution, key roles of community health officers, and the positive contributions of community involvement to maternal healthcare. The inclusion of detailed data on the outcomes of maternal health programs, such as increased antenatal care and skilled deliveries, effectively highlights the model's successes. However, the review also presents notable weaknesses, primarily in addressing challenges such as financial constraints, inadequate staffing, and insufficient medical supplies that continue to hinder the model's full implementation. While the review touches on important sociocultural barriers, there is limited exploration of how these factors are directly addressed in specific communities. Furthermore, while the review provides a broad look at the role of various stakeholders, it could benefit from a deeper discussion on the contextual factors influencing the model's effectiveness across different regions in Ghana. This review was limited by the availability of recent, region-specific empirical data and the exclusion of non-English studies. Future research should focus on evaluating the differential impact of CHPS across Ghana's ecological zones and exploring digital innovations in community maternal care. Moreover, national strategies should prioritize CHO retention and context-specific adaptations of the CHPS model to improve its effectiveness and sustainability across diverse settings. Also a more detailed exploration of how regional and contextual factors such as geographical location, infrastructure, socio-cultural dynamics, and resource distribution affect the CHPS model's effectiveness across different regions in Ghana would offer a clearer picture of the model's challenges and potential solutions. In some areas, cultural resistance to healthcare delivery methods and the unavailability of resources may significantly affect the acceptance and success of the model. These factors are not always uniform across the country, which may explain the variations in the model's impact.

## Conclusion

The Community-Based Health Planning and Services (CHPS) model plays a crucial role in strengthening maternal healthcare in Ghana by improving access through community engagement and localized services. Despite its success, challenges such as inadequate funding, staff shortages, socio-cultural barriers, and political interference persist. Addressing these issues requires increased resource allocation, policy support, and collaboration between healthcare providers, communities, and local leaders. Leveraging modern technology and sustaining CHPS will enhance maternal healthcare outcomes, reduce maternal mortality, and ensure equitable access to quality care, especially in rural areas.
